# Global warming and testis function: A challenging crosstalk in an equally challenging environmental scenario

**DOI:** 10.3389/fcell.2022.1104326

**Published:** 2023-01-16

**Authors:** Luca De Toni, Federica Finocchi, Kenda Jawich, Alberto Ferlin

**Affiliations:** ^1^ Department of Medicine, Unit of Andrology and Reproductive Medicine, University of Padova, Padova, Italy; ^2^ Department of Biochemistry and Microbiology, Faculty of Pharmacy, Damascus University, Damascus, Syria; ^3^ Department of Biochemistry, International University for Science and Technology, Daraa, Syria

**Keywords:** spermatogenesis, steroidogenesis, heat exchange, birth rate, heat stress

## Abstract

Environmental pollution, accounting for both chemical and physical factors, is a major matter of concern due to its health consequences in both humans and animals. The release of greenhouse gases with the consequent increase in environmental temperature is acknowledged to have a major impact on the health of both animals and humans, in current and future generations. A large amount of evidence reports detrimental effects of acute heat stress on testis function, particularly on the spermatogenetic and steroidogenetic process, in both animal and human models, wich is largely related to the testis placement within the scrotal sac and outside the abdomen, warranting an overall scrotal temperature of 2°C–4°C lower than the core body temperature. This review will provide a thorough evaluation of environmental temperature’s effect on testicular function. In particular, basic concepts of body thermoregulation will be discussed together with available data about the association between testis damage and heat stress exposure. In addition, the possible association between global warming and the secular decline of testis function will be critically evaluated in light of the available epidemiological studies.

## Introduction

The continuous and progressive increase in the environmental temperature from the beginning of the industrial era, universally known as “global warming,” is a concept formally acknowledged in early 1990s at the international scientific level and streamlined with the United Nations Framework Convention on Climate Change, whose first edition took place in Rio de Janeiro in 1992 (Rowbotham, 1995). Environmental, agricultural, economic, social, and health consequences of global warming are currently major areas of investigation worldwide, and the strategies aimed to stem the phenomenon are among the most debated topics, supported at the national agencies and implemented at the industrial level (Rogelj et al., 2015).

Among all the medical health issues related to global warming, the effects on male fertility and testis function are undoubtedly among the most felt matters of concern (Whitmee et al., 2015). The peculiar developmental process during embryonic life, the anatomical characteristics, and the dependence on a central endocrine axis make the testis an organ particularly susceptible to external agents. It is, in fact, known that exposure to thermal shock can heavily affect spermatogenesis, a primary function of the testis (Kim et al., 2013). Nonetheless, it is difficult to establish to what extent environmental temperature variations can affect the function of this organ in homeothermic mammals, such as humans, which, by definition, has the ability to thermoregulate in response to external stimuli. This uncertainty is made even more difficult by the lack of coherence of the study models, since the levels of exposure and the clinical outcome of reference are not always unanimously defined (Walsh et al., 2019).

In this review, the influence of the environmental temperature on testicular function will be evaluated: starting from the early concepts of physiology on body and testis thermoregulation to the known molecular aspects of the regulation of testicular function by temperature and concluding with the evidence and limits shown by the most recent epidemiological-association studies.

### Global warming and testis function: A matter of concern

Fertility is a major component of the individual fitness and a central determinant of a population’s growth and persistence (Walsh et al., 2019). The most recent data worldwide indicate that the 2020 fertility rate, i.e., the average number of children per woman of childbearing age, is equal to 2.4, a value almost halved compared to that of 5 in 1960 (Data World Bank, 2021). If this trend has been widely recognized in Western countries, the most impressive fact is that even in the African continent there has been a progressive decline in fertility rates, including countries with a traditionally high generational turnover such as Niger, Somalia, Congo, Mali, and Chad (Data World Bank, 2021). This evidence is the result of multiple factors of geo-political, social, economic, biological, and, no less important, environmental nature. The interaction between the last two factors has drawn attention in terms of clinical and experimental investigations. In this context, a key role is attributed to the environmental influence on the quality of semen in humans. In fact, although not unequivocally conclusive about the chances of parenthood, semen parameters are recognized as a major descriptor of male fertility potential and a marker of the male health status (Boulicault et al., 2021). However, even taking into account non-pathological variability of sperm parameters across populations, there is a common belief the quality of human semen is progressively declining over time. In numerical terms, a systematic review and meta regression analysis by Levine et al. involving 185 studies that accounted complexively 42,935 men from unselected Western countries, estimated a variation of −0.70×10^6^ cells/mL per year in sperm concentration and −5.33×10^6^ cells per year in total sperm count/ejaculate over the period of time between 1973 and 2011 (Bahadur G et al., 1996; Levine et al., 2017). An exhaustive discussion of this topic, highlighting major strengths, criticisms, and claims of a possible causal relationship between sperm count, lifestyle, and environmental factors is provided in the review by Auger et al. (2022). In any case, a strong parallelism is evident between the environmental modifications associated with human activities following the industrial revolution and the variations in the seminal parameters documented in the same period of time (Auger et al., 2022; Damon Matthews and Wynes, 2022). The global nature of climatic warming, therefore, raises doubts about the possible role of ambient temperature as an environmental interferent on the quality of human semen as an index of testis function.

The study of the impact of temperature on fertility, as a high expression of developmental biology, is intimately affected by methodological biases that are difficult to address. As underpinned by Walsh et al. (2019), the effect of environmental temperature on human health should be investigated through a toxicological approach, creating dose-response curves able to define precise thermal fertility limits, above and below at which a species, particularly humans, loses fertility (Lutterschmidt and Hutchison, 1997). Most importantly, the exposure to static or fluctuating temperature stress should be taken into consideration, in order to simulate real life-thermal stresses of natural populations. The resulting fertility reaction normograms would represent adequate tools for estimating the loss of reproductive performance under certain environmental conditions. Anyway, beyond the ethical issues of this approach, the direct measure of the offspring production often results unfeasible, as in the case of species with extremely slow generation times. For this main reason, representative bio-markers are chosen for their correlation with the reproductive performance, as is the case with semen parameters. However, there is no complete bi-univocity between the abnormality of the seminal parameters and the worsening of the fertility outcome (Guzick et al., 2001). To date, as discussed below, the effect of environmental temperature on the function of the testis is based on animal experimental models, well defined methodologically but not always translatable to humans, human models of occupational exposure and epidemiological studies where, however, it is lacking yet a precise characterization of the thermal stress linked to environmental conditions.

### The evolutionary origin of the external testis in humans and its thermoregulation mechanisms

The placement of the testes in humans (and in other homeothermic mammals), anatomically external to the abdominal cavity, betrays the functional dependence of this organ on temperature. The scrotal detachment of the testis from the lower abdomen, in fact, provides an average tissue temperature of about 2°C–4°C lower than the core body temperature (Mieusset and Bujan, 1993). Whether the driving force of this anatomical displacement has been the achievement of better reproductive fitness, linked to a lower internal organ temperature, or that the lower temperature of the testicle is a physiological characteristic derived from the evolutionary advantage of having an external gonad is currently unclear and a matter of investigation. Indeed, compared to the original abdominal location of the testis common to all animals, the evolutionary descend to a variable extent to their final mature location is the result of a specific selection pressure, having the water–land transition and the endothermy affirmation as major influences, further harmonized by specific intra-tissue cell crosstalk (Miller and Torday, 2019). The most complete hypotheses available to date suggest that the evolutionary water–earth transition on one hand favored a greater availability of oxygen from the atmosphere. On the other hand, it required a substantial adrenergic boost to sustain blood pressure for ground living and movement (Volkmann and Baluška, 2006). As a consequence of the increased adrenergic tone, the derived increased lipolysis from the adipose tissue allowed a greater availability of energy fat substrates, favoring the endothermic regulation of body temperature (Lawson et al., 1978; Celi et al., 2015). It should be noted that animal homeothermia itself constituted an evolutionary advantage, rendering not necessary an arsenal of metabolic enzyme isoforms, with different optimum working temperatures, typical of heterothermic organisms (Rodman and McHenry, 1980). In this context, a variable degree of testis externalization represented an evolutionary tissue response to specific biochemical challenges such as the lowering of intra-abdominal pressure peaks, due to transient blood pressure increase associated with hunting/fighting/escape activity, or to reduce the occurrence of temperature-dependent mutagenic events (Lovegrove, 2014). Collaterally, the maturation of germ line cells in a lower-temperature environment allowed the development of temperature-sensing properties by spermatozoa such as temperature-driven migration, known as *thermotaxis* (Xiao W. et al., 2022). This functional property of spermatozoa appears pivotal during the fertilization process by supporting the migration of male gametes though the female genital tract from the uterine isthmus, where sperms are deposited upon ejaculation, to the tubal ampulla where oocyte fertilization is likely to take place. Importantly, a physiologic temperature gradient of 2°C–4°C exists between the isthmus and ampulla, representing both a migration drive for sperms cells and a selection screen for functionally competent cells (Bahat et al., 2003).

In addition to the external anatomical location, a key aspect is the maintenance of the reduced testicular temperature by the thermoregulation of the organ, characterized by both common aspects and differential elements compared to other body organs. Deferring a more in-depth discussion to more authoritative reviews on the topic (Cramer et al., 2022), body thermoregulation can be defined as all those systems aimed at maintaining tissues at relatively constant values of temperature. In practice, this translates into the typical maintenance at 36°C–38°C of the central tissues of the body such as the head and thorax. Although body temperature is a critical parameter, being associated with the correct protein and cellular function, the extent and tolerability of its oscillations are subject to some degree of elasticity depending on the particular conditions in which they occur. For example, cell temperatures of 40°C–45°C associate with protein denaturation and cell death (Lepock, 2003) and core body temperatures greater than 40°C associate with an increased risk of heat injury and heat stroke (Bouchama and Knochel, 2002). However, during a sports performance, peak body temperatures over 41°C are almost harmless and increasingly tolerated with the athlete’s thermal acclimatization and aerobic training (Racinais et al., 2019). In general, there can, therefore, be distinguished a “heat stress,” resulting from a passive transfer of heat load from the environment to the individuals, and a “heat strain” resulting from the increased metabolic activity and heart rate of the individual. In basal and resting conditions, the energy balance around a subject has metabolic activity as the main energy input of the system, whilst heat transfer *via* conduction, radiation, convection, and evaporation from the skin surface and/or respiratory tract represents major heat loss. Conditions that associate with a rate of metabolic heat production greatly exceeding the total heat loss result in body heat accumulation and rise of the core body temperature. This is the case of hot and humid environments, that can be even more exacerbated by factors that worsen the heat exchange efficiency, such as high air temperature or high mean radiant temperature, ambient vapor pressure, air velocity, and the presence of insulating clothing (Kenny et al., 2017). The autonomic response to heat stress is mainly expressed through the establishment of a reflex circuit in which the brain is possibly the main thermosensitive organ and the main integration center of thermo-afferent stimuli at the same time (Barbour, 1921; Nakamura and Morrison, 2010). The sensing of temperature by the skin, although important, is considered to have an auxiliary role, more involved in the response rate of the thermoeffector output (McCaffrey et al., 1979). In humans, the latter can be evaluated through the postganglionic skin sympathetic nerve activity (SSNA) in peripheral nerves (Greaney and Kenney, 2017), responsible for cutaneous vasodilation and sweating during whole-body passive heat stress (Barry et al., 2020; Greaney et al., 2021). The occurrence of both these events maximizes body surface heat loss by sweat evaporation (Smith and Havenith, 2011). Available studies aimed to address the relationship between multiunit SSNA bursts and thermoeffector output used hot water-perfusion suits to simulate changes in ambient temperature. A negligible activation of the SSNA at low levels of heat stress has been shown (ambient temperature ∼24°C), suggesting major local rearrangement of cutaneous blood flow due to the removal of the vasoconstrictor stimulus (Nordin, 1990). Active cutaneous vasodilation and sweating through the engagement of SSNA is observed at increasing levels of heat stress (up to 47°C, Kamijo et al., 2011). It remains currently to be clarified whether active cutaneous vasodilation and sweating are controlled by a separate or unique pool of postganglionic neurons. In general, at ambient temperatures greater than 34°C, lacking a favorable thermal gradient for dry heat toward the environment, the evaporation of sweat becomes the only means of heat loss from the body.

**FIGURE 1:**
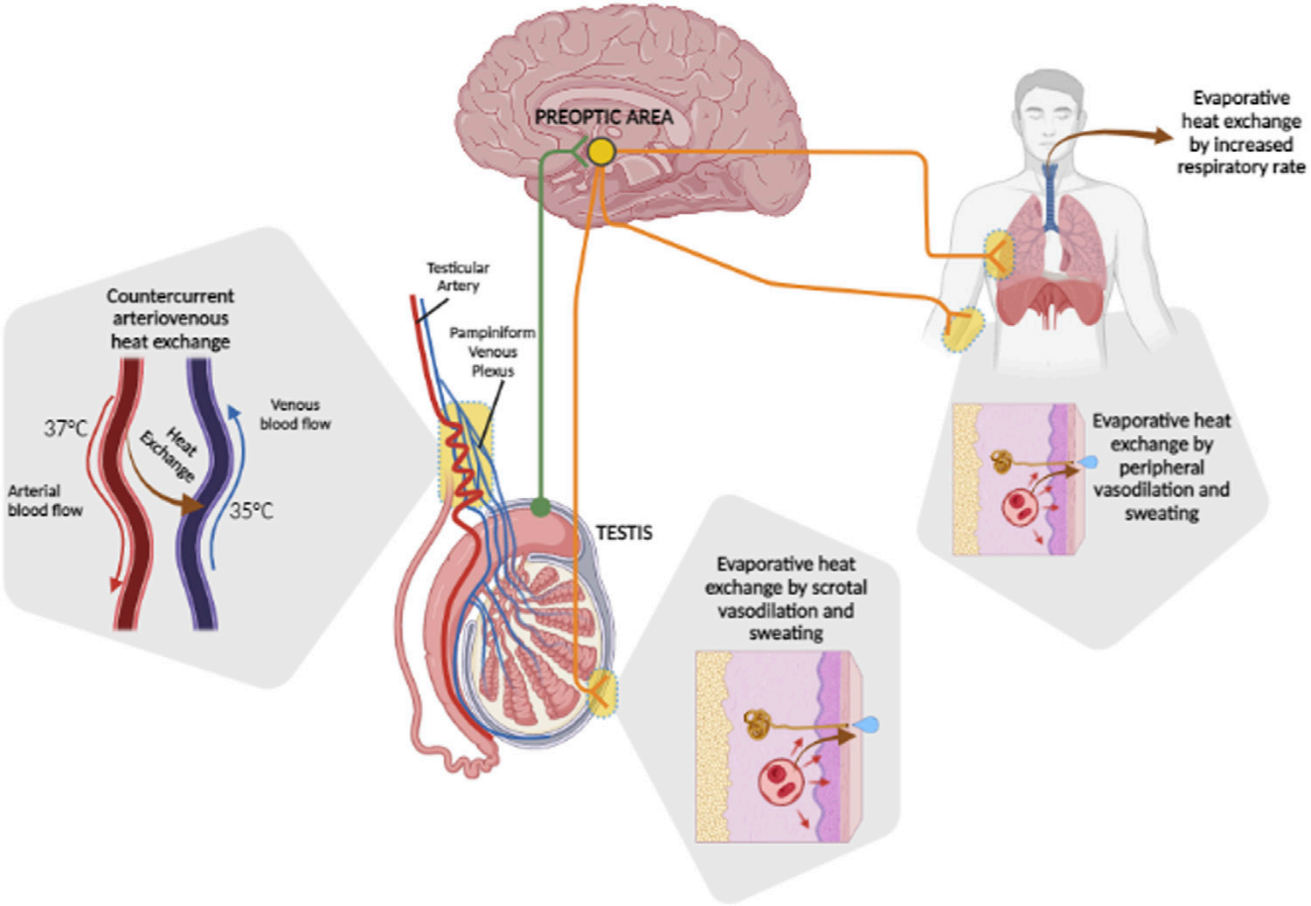
Heat dissipation mechanisms of the testis. Basal testis temperature is maintained at 2°C–4°C below the core body temperature by countercurrent arteriovenous heat exchange between the incoming testicular artery, which carries blood at ∼37°C, and the outgoing pampiniform plexus, carrying blood at ∼35°C. Variations of tissue temperature are locally perceived by nerve afferents of the preoptic area of the brain (green fibers) which, depending on the entity of heat challenge, integrates thermoregulatory response (orange fibers). These may rely on local evaporative heat exchange by scrotal vasodilation and sweating, which can be extended systemically, eventually involving evaporative heat exchange by an increased respiratory rate.

In this context, the thermoregulation system of the testis is characterized by some peculiarities compared to the systemic one (Figure 1). These are essentially provided by the arterio-venous architecture at the inguinal canal. In fact, the testicular artery in entry presents a series of recurring loops in close contact with the exiting pampiniform venous plexus, allowing an important heat exchange surface between the two vascular systems. The inlet arterial blood flow, being a direct dependence of the superior mesenteric artery, owns a temperature close to that of the core body, 37°C. On the other hand, the outgoing venous blood flow has an average temperature of 35°C. Such a combination of a temperature gradient and contact surface results in countercurrent heat exchange, according to which arterial blood achieves the testicular tissue with a lower temperature than the systemic circulation (Tritto, 1991). Experimental evidence of this anatomic model was provided by Silva et al. in water buffalo bulls, a mammal with external genitalia comparable to humans (Silva et al., 2018). The authors aimed to evaluate the scrotal surface temperature patterns and semen quality of buffalo bulls in relation to seasonal variations of environmental temperature and humidity, through the measurement of the scrotal temperature of the animal by infrared thermography. It was possible to verify the progressive reduction in the scrotal temperature from 34°C at the spermatic-cord level, to 31°C at the epididymal-tail level, supporting massive heat dissipation at the proximal portions of the scrotum. However, the regulation of testicular temperature shows to be more complex involving, in addition to scrotal vasodilation and sweating (Song and Seo, 2009), systemic reflex reactions. In the early 1960s, Waites et al. evaluated the reflex responses from scrotal warming in rams, through the application of a testis thermostatic water-bath. In the face of insignificant thermoregulatory responses associated with skin warming of the trunk at 36°C, the increase in scrotal skin temperature from 32°C to 36°C was associated with an increase in the respiratory rate up to over 200 breaths per minute, following which there was a progressive reduction in the core body temperature of 2°C. Polypnea disappeared upon wool shaving and maintenance of surface body temperature below 35°C. In addition, it has been shown that the increase in scrotal temperature to 35°C in shaved rams was associated with waves of scrotal sweating at intervals of 2–14 minutes accompanied by local temperature drops of 2.6°C. On the other hand, in the unshaven animal, fluctuations in scrotal temperature alternated with an increased respiratory rate (Waites, 1961; 1962; 1963). All these thermoeffector responses were abolished by the local administration of an anesthetic at the level of the superior perineal nerves (Waites, 1963), supporting a major role of central processing of the thermo-sensory input from the scrotum.

Considering the major role of blood circulation in regulating the temperature of the testis, it is not surprising how vascular disorders of this organ may result in its altered thermoregulation. One example among all is varicocele. Varicocele associates with a loss of the containment properties of the venous valves within pampiniform plexus, giving rise to a venous stasis that hydrostatically weighs on the testis. Importantly, varicocele can be perceived during common diagnostic maneuvers such as abnormal dilation and tortuosity of the pampiniform plexus venous (Macey et al., 2018). The reduction in venous drainage linked to varicocele worsens the efficiency of countercurrent heat exchange with arterial inflow through the testicular artery, resulting in an increase in scrotal temperature consistent with the clinical scoring of varicocele. Early evidence dates back to the late 1990s, when Wright et al. showed that scrotal temperature in varicocele patients was nearly 1°C higher than that observed in control subjects and that varicocele correction through microsurgical varicocelectomy, was associated with a significant improvement in testis thermoregulation (James Wright et al., 1997). In 2015, data from our group confirmed and expanded this evidence by the use of a portable device for 24 h-monitoring of the skin temperature, and in particular, the scrotal temperature (Garolla et al., 2015). In fact, the opportunity of continuous monitoring allowed the identification of physiological fluctuations in the scrotal temperature, in control subjects, with circadian-like frequencies as previously suggested by Hjollund et al. (2002). In varicocele patients, the extent of these oscillations was strongly reduced and associated with a significantly higher mean scrotal temperature than in controls, highlighting a vascular-based thermal-regulation of the testis which is more complex than that detectable by punctual and static evaluations.

### Impact of heat stress on testis function: Evidence from experimental models

Testis function is the result of a proper interplay among the different cell populations constituting this peculiar tissue. There can be distinguished cells of the seminiferous tubule, the mono-stratified epithelium of Sertoli cells that harbor the differentiating germ cell lines during the spermatogenesis process, and the intertubular interstitium where the Leydig cell population is primarily involved in the steroidogenetic function (Wakayama et al., 2022). Abundant literature, aimed at identifying the effects of thermal stress on the testis cell populations, has been provided.

Adjacent Sertoli cell membranes form tight junctions that constitute the blood–testicular barrier, a specialized anatomical barrier warranting a protected environment for germ cell development (Dym and Fawcett, 1970). In turn, tight junctions are a particular example of occluding junctions formed by three classes of integral membrane proteins: occludins, claudins, and junctional adoption molecules (Mruk and Cheng, 2004). Occludin itself plays a key role in tight-junction formation and, since *occludin* ^*−/−*^ mice show severe seminiferous tubule atrophy and major loss of the germ cell component, it is thought to play an important role in male fertility (Saitou et al., 2000). On the other hand, ZO-1 is responsible for the assembly, scaffolding, and regulation of transmembrane tight-junction proteins by binding both tight-junction proteins and cytoskeletal actin, whilst caludin-3 is considered a tight-junction protein that occurs during the formation of the blood–testicular barrier (Stevenson and Keon, 1998; Meng et al., 2005). Cai et al. (2011) showed that the expression of the tight junction components occludin, claudin-3, and zonula occluden-1 (ZO-1) in mice was decreased for 24–48 h after scrotal heat exposure to 43°C for 30 min, returning then to normal levels after 3 days.

In a pioneer study conducted by Rockett et al. (2001), a model of heat stress was applied to rodents by submerging the lower end of the animals in a water-bath of 43°C for 20 min. After 8 hours of exposure to a 43°C water-bath, diffuse vacuolar degeneration was observed throughout the seminiferous tubule cell populations, with the nuclei of the germ cells showing highly condensed chromatin and diffused detection of apoptotic bodies. After 16 h of thermal stress, germ cell degeneration proceeded through the generation of giant cells of spermatocyte origin. In addition, apoptosis-dependent cell DNA fragmentation was observed in cells undergoing spermatogenesis arrest and vascular degeneration (Rockett et al., 2001). Almost similar findings were observed nearly 20 years later. A recent study by Hirano et al. evaluated the extent of temperature-dependence of spermatogenesis in murine models (Hirano et al., 2022). An artificial condition of cryptorchidism was obtained in C57BL/6 J mice by wrapping testes into epididymal fat pads and positioning the artifact in the abdomen. The continuous monitoring of the scrotal temperature was obtained through the implantation of a transponder in the scrotal sac and the peritoneal cavity. The authors observed that, compared to the physiological murine scrotal temperature of 34°C, spermatogenesis had not progressed beyond the primary spermatocyte, late spermatocyte, or round spermatid stages at a testis temperature of 38°C, 37°C, and 36 C, respectively,. At the core body temperature of 38°C testis cytology showed only the presence of spermatocytes with no detection of round cells or elongated spermatids. *Ex vivo* cultures of testis tissues at different temperatures showed that specific markers of Sertoli cells and spermatogonia were abundantly present at both 34°C and 38 C. However, at 38°C, spermatocytes were unable to complete chromosomal pairing, resulting in the triggering of apoptosis (Hirano et al., 2022).

In addition to rodents, the testis effect of heat stress has been evaluated in other types of mammals. Recently, Garcia-Oliveros et al. evaluated the impact of environmental temperature on bull spermatozoa (Garcia-Oliveros et al., 2020). In this case, the heat stress model was applied through a thermostated scrotal bag maintained at ∼32°C, compared to ∼30°C of controls, for 96 h. After 14 days of thermal stress, the lipid membrane peroxidation levels of spermatozoa showed a significant increase, whilst the assessment of mitochondrial membrane potential, cell motility parameters, and the plasma membrane integrity of sperm cells showed a significant decrease compared to the control group. After 28 days of heat stress, the sperm DNA fragmentation index also showed a significant increase as a possible late effect (Garcia-Oliveros et al., 2020).

In 2020, Wu et al. aimed to investigate the molecular fingerprint of human spermatozoa exposed to acute heat stress through a proteomic approach (Wu et al., 2020). Testicular warming at 43°C, administered through sessions of 30-min water bath/day for 10 consecutive days, was associated with the deregulation of more than 60 proteins at 6 weeks after the heat stress, most of which were involved in spermatogenesis, fertilization, or other aspects of reproduction. The authors also found a similar pattern of deregulation upon the application of the same heat stress model in mice. In particular, significant downregulation was observed for the protein of A-kinase anchor protein 4 (AKAP4), involved in sperm motility and acrosome reaction, the cytoskeleton proteins’ outer dense fiber protein 1 (ODF1), testis-specific glyceraldehyde-3-phosphate dehydrogenase, (GAPDHS), sperm equatorial segment protein 1 (SPESP1), and actin-related protein T2 (ACTRT2) (Cheng et al., 2014; Wu et al., 2020). On these bases, the authors argued that the deregulation of these proteins, closely associated with the reversible decrease in sperm viability and concentration, was mainly driven by heat stress (Wu et al., 2020).

Many of the studies performed in humans aimed to identify specific pathological and lifestyle conditions associated with the increase in scrotal temperature and the related impact on testis function (de Fleurian et al., 2009). As an example, one of the most recognized factors that contributes to the increase in testicular temperature is the posture of the subjects, such as the maintenance of a prolonged sitting or lying down pose (Brindley, 1982). Tight underwear has also been suggested to affect scrotal temperature by limiting testis movement and air circulation; however, no statistically significant effects have been observed at the seminal level (Munkelwitz and Gilbert, 1998; Hjollund et al., 2002). Even working with the laptop resting on the lap, close to the genital area, has been associated with a worsening of seminal parameters (Sheynkin et al., 2005). To this regard, occupational exposure to radiant heat sources, representing the most part of the day in a timely point of view, significantly affects the increase in scrotal temperature (Durairajanayagam et al., 2015). Other studies aimed to evaluate the effect on human testis of a specific model of heat stress, such as the immersion of the body in hot baths, whirlpools, or water heated to temperatures above 37°C. In general, wet testis hyperthermia has a reversible negative effect on sperm motility (Shefi et al., 2007). In a very recent study, Fraczek et al. evaluated sperm parameters, sperm DNA integrity, and major markers of oxidative stress, in subjects who voluntarily underwent a semen test and completed a lifestyle questionnaire (Fraczek et al., 2022). Compared to controls, study participants reporting prolonged genital heat stress, namely, professional drivers and infertile men with varicocele, showed that the total sperm antioxidant capacity was reduced (Fraczek et al., 2022). On the other hand, the catalase activity and sperm DNA fragmentation both increased (Fraczek et al., 2022).

It is widely accepted that redox imbalance is a common mechanism underlying the pathophysiology of male infertility, thus representing a major risk factor in male fertility disorders. Reactive oxygen species (ROS), including hydrogen peroxide (H_2_O_2_), superoxide anion (O_2_^−^), nitric oxide (NO), and hydroxyl radicals, are highly reactive oxidizing agents. Normally, in semen, the rate of ROS production is counterbalanced by the complex of cell antioxidant systems. In a physiological scenario, ROS are produced at low levels and play a role as mediators in key sperm processes such as capacitation, motility hyperactivation, and acrosomal reaction (Aitken, 2017). Any imbalance in the cell redox system resulting in net ROS over-production exposes cells to the negative consequences of oxidative stress such as lipid peroxidation, DNA damage, and cell apoptosis as a late event (Agarwal et al., 2003). Heat stress-related testicular damage has been frequently associated with the triggering of oxidative stress processes. In animal studies, the heat-induced oxidative stress response in the gonads finally associates with germ cell apoptosis, cell cycle arrest, and disturbances in the oxidative stress-scavenging system (Zhang et al., 2012; Park et al., 2017). In spermatozoa, the main effects associated with the increase in temperature are represented by the increase in ROS production by mitochondria, impairment of the mitochondrial membrane potential, and changes in the plasma membrane composition through the involvement of heat shock proteins. An impairment of DNA synthesis, which leads to changes in gene expression and signal transduction in germ line cells, has also been observed (Shiraishi et al., 2010).

In addition to the process of spermatogenesis, the application of heat stress to the testis has been associated with the impairment of steroidogenesis in several experimental models. Alves et al., in 2016, applied testis heat stress to rams through 72 h of scrotal insulation using a specific bag that limited heat dissipation (Alves et al., 2016). Scrotal insulation resulted in the average increase in scrotal temperature by 2°C, compared to control animals, and normal scrotal temperature was restored after 24 h from insulation removal. In addition to the impairment of sperm motility and morphology parameters after 21 days of heat stress, serum testosterone (T) showed a significant reduction in rams which underwent scrotal insulation, compared to controls (Alves et al., 2016). Aiming to investigate the effect of heat stress on the testicular function, Shadmehr et al. exposed 7–9-week-old male mice to testis heat stress by the immersion of the lower half of the body in water at 42°C for 30 min for a 15 day-period (Shadmehr et al., 2018). Compared to control animals, heat stress exposure was associated with the impaired maturation of the germ cell population, a reduction in the testicular weight and epididymal sperm parameters, and increased levels in antioxidative enzymes. From a molecular point of view, an abnormal elevation of cell regeneration markers was observed, such as the proliferating cell nuclear antigen and Cyclin D3, together with the inadequate synthesis of T. The suggested mechanism mediating germ cell damage was related to the apoptosis of germinal cells, autophagy, DNA damage, and increased generation of reactive oxygen species in testes (Shadmehr et al., 2018). Almost overlapping results were reported in a recent study conducted by Jeremy et al., aiming to examine the effect of leptin or its synthetic analog, on heat-induced testicular impairment in mice (Jeremy et al., 2022). Interestingly, a single heat stress treatment at 43°C, obtained by submerging the lower half of the body in a thermostatic water bath for 15 min, was associated with significantly reduced germ cell proliferation and decreased circulating levels of T. Data from Li et al. focused more specifically on the effect of heat stress on the Leydig cell population (Li et al., 2016). In this study, adult rats underwent testicular heat exposure by single water bathing at 43°C for 30 min, which was sufficient to induce Leydig cell hyperplasia. In addition, proliferating Leydig cells showed an increased expression of cell cycle proteins, and a reduction of both serum and testicular T concentrations were observed. This was associated with poor T biosynthesis by the suppressed expression of steroidogenic enzymes, including cytochrome P450 family-17 (CYP17) and the steroidogenic acute regulatory protein (StAR). To this regard, a recent paper by Rizzoto et al. showed a significant upregulation of the *StAR* gene 14 days after the administration of heat stress by single exposure to 40°C for 20 min in C57BCL/6 elite male mice (Rizzoto et al., 2020). This finding was interpreted as a compensating response to a demand of increased T support for sustaining germ cell renewal (Rizzoto et al., 2020).

The effect of heat stress on T production has also been investigated in human models. In addition to the exposure to chemical agents, such as heavy metals, chemical agricultural products, solvents, and physical agents, such as noise and vibrations, radiation and environmental heat have also been accounted as occupational risk factors influencing testis function (Lähdetie, 1995; Thonneau et al., 1998). Aiming to investigate the effect of heat stress exposure, evaluated by the wet bulb globe temperature (WBGT), on the levels of sex hormones in foundry section workers, Thonnneau et al. found that nearly 43% of workers suffered from low free T-levels (Thonneau et al., 1998). Another factor associated with heat stress administration to the testis is prolonged sitting in the car, which results in an increase in the scrotal temperature up to 2°C after 2 h of sitting (Bujan et al., 2000). In a study conducted on taxi drivers in Rome, Italy, evaluating T-level and sperm parameters in relationship to the length of service, mean salivary T-levels did not vary significantly with years in the profession, although the percentage of spermatozoa with normal morphology was significantly lower in taxi drivers than in the controls (Figà-Talamanca et al., 1996). This finding was even more pronounced in those drivers who had been working for a long time (Figà-Talamanca et al., 1996). Finnish sauna, which is frequently used for recreational purposes, also represents a good model of total-body heat stress, useful to show that the testis correlates to hyperthermia. It has been documented that daily exposure to a sauna for 2 weeks may affect male fertility, particularly, the impairment of sperm motility parameters (Saikhun et al., 1998). In 2013, Garolla et al. investigated the effect of the sauna on human spermatogenesis in 10 healthy subjects who underwent two sessions per week for an overall period of 3 months (Garolla et al., 2013). Authors documented no significant change in serum levels of sex hormones in spite of a strong, though reversible, impairment of sperm count and motility together with changes in mitochondrial function, chromatin protamination and sperm DNA condensation (Garolla et al., 2013).

#### Evaluating the effect of global warming on human health: Not just a matter of heat stress

That the general trend toward the progressive increase in the average environmental temperature is directly associated with anthropogenic activities is now clear and irrefutable (Masson-Delmotte et al., 2021). There have been identified anthropogenic drivers of climate change, among which CO_2_ emissions surely represent the major gas responsible for the greenhouse effect (Masson-Delmotte et al., 2021). However, other drivers such as other non-CO_2_ greenhouse gas and aerosol emissions have been identified (Lelieveld et al., 2019). In mechanistic terms, the role of greenhouse gases in environmental temperature increase has been modeled since the mid-1800s (Tyndall, 1861). Briefly, approximately 30% of solar electromagnetic radiations are reflected to space by clouds, dust, and haze (Ramanathan and Feng, 2009), whilst the remaining 70% penetrates the atmosphere and reaches Earth’s surface. Here, incident radiation with a wavelength within the UV-Visible range is absorbed, contributing to ground surface warming through the release of infrared (IR) radiations in all directions of space. Part of these IR radiations re-emerge from the atmosphere, while part are retro-reflected by the atmosphere itself, contributing to the heating of the lower atmospheric layers. The amount of this retro-reflection is greater, and the higher is the atmospheric content of the aforementioned greenhouse gases (Huber and Knutti, 2012). The result is the direct association between the percentage of atmospheric CO_2_ levels and the average environmental temperature of the biosphere (Damon Matthews and Wynes, 2022). The specific treatment of these topics is thematic in a separate scientific disciplinary sector and goes beyond the scope of this review; however, from an absolutely analytical point of view, for the purpose of the impact of global warming on health, we can ideally distinguish the effects of the two main phenomena linked to global warming: the increase in average environmental temperature and the increase in the frequencies of heat waves (Perkins et al., 2012; Haustein et al., 2017). The first is defined as the increase of the mean environmental temperature compared to the 1850–1900 baseline, currently established as +1.25°C (Haustein et al., 2017). The second still does not find a consensus in terms of a definition and is generally considered as temperatures that are either unusually high compared to characteristic local environments or extend to the level which may harm human health and infrastructures (Awasthi et al., 2022). Given the “extraordinarily anomalous” nature and the temporal limitation, it is relatively easier to analyze the health consequences derived from exposure to a heat wave, being able to establish with sufficient precision the conditions prior to the climatic event, the extent of this last (temperatures reached, degree of humidity, duration of time, etc.) and the type, severity, and epidemiology of the immediately following clinical events. As an example, heat waves recorded in France in 1983 and 2003 were associated with more than 300 deaths in the sole city of Marseille and 15,000 deaths in the overall France territory, respectively (Cadot and Spira, 2006; Fouillet et al., 2006). In the summer of 1995 in Chicago, 365 deaths among the elder population were recorded, whilst more recently in 2018, a 7 day-heat wave recorded the hospitalization of 35,000 people in Canada and 80 deaths in Japan (Pörtner et al., 2022). Globally, the increased mortality rates were directly associated with peak temperatures, such as heatstroke, hyperthermia, and dehydration, or complications to major cardiovascular, respiratory, and neurological diseases (Pörtner et al., 2022). In the case of heat waves, fatalities appear to be mainly ascribable to heat stress associated with the exposure to an extreme temperature or to compensatory inability in a pre-existing pathological condition.

**FIGURE 2:**
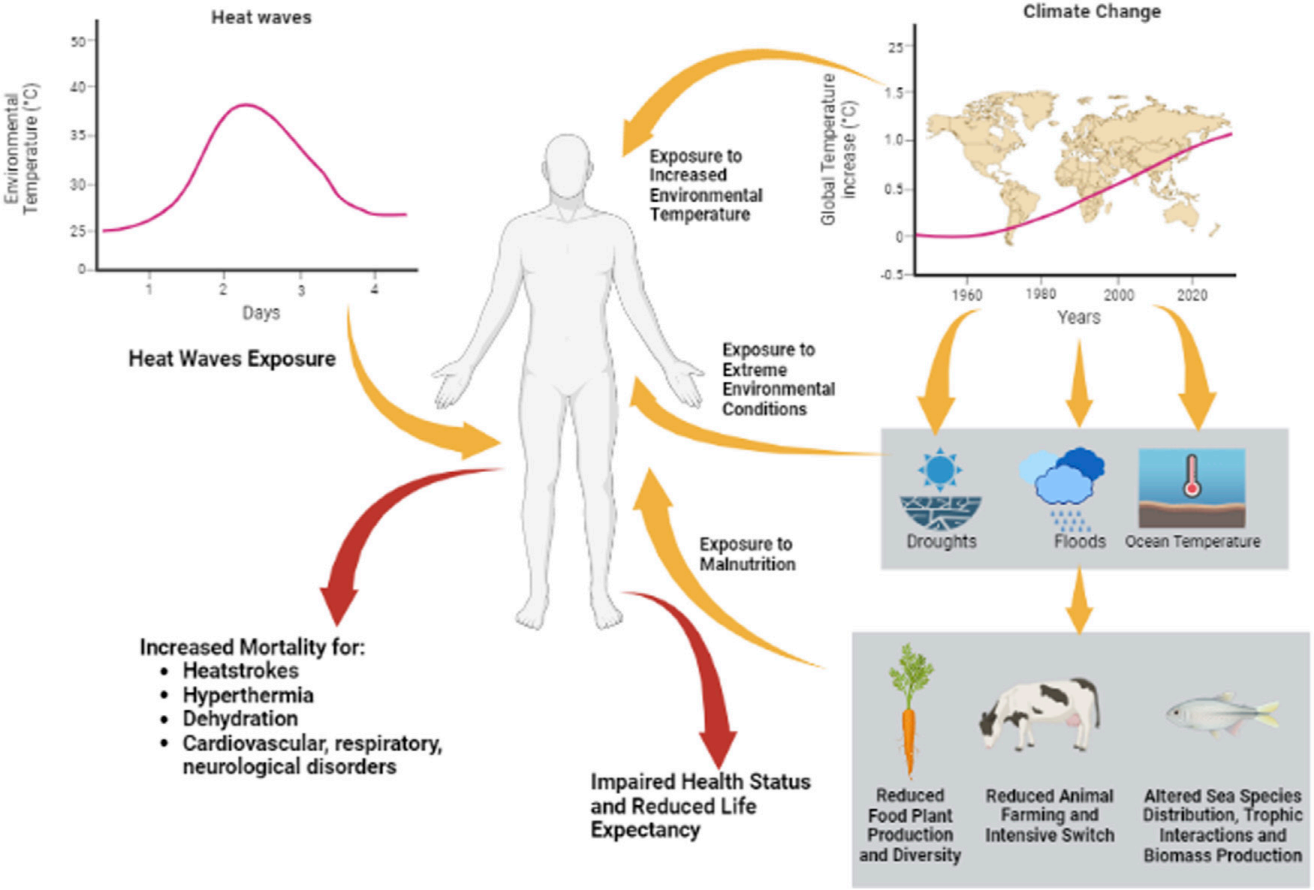
Heat challenges can be summarized as heat waves, generally considered as temperatures that are either unusually high compared to characteristic local environments, and/or climate change through the increase in average environmental temperature compared to the 1850–1900 baseline. The first have short-term effects on human health, increasing the risk of mortality for heat strokes, hyperthermia, dehydration, and cardiovascular, respiratory, or neurological disorders. The second has more complex effects on the health status and life expectancy of humans, involving increased exposure to extreme environmental conditions and exposure to malnutrition for impairment of the biosphere.

On the other hand, the progressive and gradual increase in the environmental temperature has a wide-ranging impact on the various environmental determinants of human health. It is, thus, much more difficult to identify the single role of temperature increase in the set of elements that it itself determines. Among the many examples, available studies underpin that climate change severely alters crop productivity, increasing the risk of drought or floods in geographical areas (Schlenker and Lobell, 2010; Lobell et al., 2011; Moore and Lobell, 2015; Ray et al., 2015; Lesk et al., 2016; Schauberger et al., 2017). With the reduction in arable land, the production of food of animal origin also gradually shifts from a free-range farm to intensive farming, reducing the quantity and diversity of foods available and increasing the risk of zoonosis (Koneswaran and Nierenberg, 2008; Rupasinghe et al., 2022). Similar or even worsened considerations can be made for seafood. Seawater has major importance as thermal flywheel, absorbing 90% of the heat load associated with greenhouse gas-global warming (von Schuckmann et al., 2020). The resulting increase in the ocean mean temperature, the reduction in dissolved oxygen concentration in the face of lowering of marine pH and acidification, severely impacts marine ecosystems (Frölicher et al., 2018), altering the species distributions, trophic interactions, and the overall biomass production (Thackeray et al., 2010; Poloczanska et al., 2016; Provost et al., 2017). From an anthropic point of view, all this translates to the decrease and changes in catch composition, as well as shifts in stock distributions (Cheung et al., 2013; Free et al., 2020). Therefore, when long-term associations with the increase of average environmental temperatures are evaluated, there are several factors that, although difficult to quantify, they cannot be excluded. These are, as an example, the increased exposure to extreme environmental conditions and risk of malnutrition, representing themselves important negative prognostic factors on the health status, including the fertility status and life expectancy in humans (Djoumessi, 2022) (Figure 2).

#### Seasonal variations of sperm parameters in relation to the environmental temperature

The influence of ambient temperature on seminal parameters, as an index of testicular function, is now confirmed by a number of epidemiological studies. As an example, a recent study on this topic is that from Kabukçu et al. (2020), who evaluated the monthly variations of sperm parameters in 4,874 male subjects of Turkish nationality, residing in Denizli province in western Turkey, recruited in the period November 2012-April 2020. Importantly, severe oligozoospermic subjects (semen sperm concentration <4×10^6^ cells/mL) and patients with varicocele were excluded from the analysis. Results showed that sperm concentration and total sperm count were significantly lower in July and August than in December, May, and June, reaching their lowest values in October. Also, the total progressive motile sperm count was significantly lower in October than in May, but no noticeable seasonal variation of sperm morphology and ejaculation volume was observed. Interestingly, sperm concentration, total sperm count, and total motile sperm count were progressively inversely correlated with the mean ambient temperature and the temperature–humidity index (THI), a multifactorial parameter which, taking into account temperature and humidity, aims to identify the environmental comfort conditions for THI values between 15°C and 20°C. Relative humidity and daily sunlight exposure showed no correlation with any of the sperm parameters.

A more comprehensive paper, taking into account other environmental interferents possibly related to temperature, is that by Santi et al. (2018), the first facing this topic through a big data analysis approach. The authors considered 5,131 men residing in the province of Modena, in northern Italy, who provided 5,573 semen samples from January 2010 to March 2016. Semen analyses were all centralized at a unique laboratory facility and the results were correlated with local environmental temperatures and air pollutant levels, such as particulate matter (PM) and nitrogen dioxide (NO_2_), monitored on geo-localization bases provided by the local environmental protection agency. The total sperm count, sperm concentration, sperm motility, and morphology data were significantly and inversely correlated with the maximum and minimum temperatures registered on the day of collection. Interestingly, the dichotomous subdivision of semen samples according to the collection during the days of extreme temperatures (below the first percentile or above the third percentile of the temperature distribution) and on the days of normal temperature (within the first and the third percentile), showed a significant difference in the total sperm count between the two groups, being greater during normal temperatures, suggesting a two-tailed distribution of the number of spermatozoa according to the environmental temperature. In addition, considering that the minimum time required for the complete maturation of a spermatozoon from the corresponding gonocyte requires at least 70–80 days (Hermo et al., 2010). The authors also correlated sperm parameters with the environmental data of the previous 30–60 and 90 days. Similar negative correlations were found between sperm parameters and average temperatures in the 30 and 60 days before collection, but no significant correlation was observed for the temperature of the 90 days before. In general, the total sperm count per ejaculation showed significantly lower values in summer and autumn compared to winter and spring, showing also an inverse relation with daylight duration. On the other hand, air PM10 and PM2.5 levels were inversely related to sperm progressive motility and the percentage of sperm cells with abnormal morphology.

Two studies on large cohorts in 2020 and 2022, respectively, essentially confirmed the inversed relation between “extreme” environmental temperatures and sperm parameters. Zhou et al. reported the results of a longitudinal study, conducted on 10,802 males from the general population residing in Wuhan (China), that lasted from March 2013 to April 2018 (Zhou et al., 2020). In the exposure-response analysis, the authors documented that all sperm parameters had an optimum ambient temperature of 13°C. A significant, and almost linear, reduction of the aforementioned parameters was observed by moving away, both positively and negatively, from this optimal temperature condition. In parallel, Xiao et al. evaluated 4,912 semen samples from 1,310 donors residing in Guangdong Province (China) (Xiao L. et al., 2022). As for the aforementioned studies, the sperm parameters of semen samples collected in spring were higher than those collected in winter. In addition, the daily average temperature standard deviation and the day-to-day maximum value of temperature variation, two indexes of ambient temperature variation, had a significant impact on the decrease in sperm concentration of semen samples collected 90 days later. Correlation was still significant even after correction for humidity and air PM contaminants.

In spite of a wide availability of studies reporting a significant association between seasonal temperature variation and semen parameters, there are currently very few studies, if not just one, that report a possible correlation between environmental temperature trends and seminal parameters over a long period of time. In 2012, Yogev et al. aimed to evaluate the mutual influence of sperm parameters of freshly ejaculated semen on sperm motility upon thawing. Importantly, authors performed a longitudinal analysis over 18 years (1992–2010), accounting the environmental temperature as a possible confounder. Together with an effective increase in the average yearly temperature by 0.09°C per year, a parallel significant decrease in sperm concentration, total count, motile, and normo-conformed fraction were documented (Yogev et al., 2012). In particular, a significant negative association was observed between the monthly average temperature and, respectively, sperm concentration, normal morphology, total count, and total motile count.

Taken together, current evidence supports the association between the season environmental temperature and sperm parameters, highlighting a likely detrimental effect of extreme temperatures. However, though significant, there is scarcity of data linking the growing trend of the ambient temperature with the decline of sperm parameters.

### Temporal trends in T-levels

In addition to the spermatogenic activity, T production is another major testis function (Gray et al., 1991). Some efforts have been spent to address the possible association between seasonal temperature variations and the pattern of sex hormones in humans, unfortunately without consistence. As an example, a study of the seasonal effects on blood T concentration in a large sample of 4,462 U.S. military veterans, documented that T-levels show seasonal peak during the colder months of the year, such as November and December (Dabbs, 1990). Also, Perry et al., in a study conducted on 65 African American participants, showed a circannual variation of both total and bioavailable T-serum levels, with a peaking trend in winter during February and March (Perry et al., 2000). Differently, in a study conducted on 16 participants in 1996, Meriggiola et al. found a significant and transient increase in T-levels in June, with essentially steady levels during the rest of the year (Meriggiola et al., 1996). More recently, Santi et al. evaluated serum T-levels through a big data approach, showing a seasonal rhythm with higher levels in summer (Santi et al., 2018). Finally, studies by Renberg et al. and Bellastella et al. in 10 healthy subject and 207 vasectomized patients, respectively, reported a peaking of T-levels in autumn (Reinberg et al., 1988; Bellastella et al., 2013). Major inconsistencies among studies are attributed to characteristics of the subjects, geographical settings, and the influence of daily sunlight exposure through the secretion of melatonin (Santi et al., 2018).

On the other hand, there is a general consensus viewing an age-related decline in serum T-levels after reaching adulthood, estimated by some authors to be around .2%–.8% per year (Gray et al., 1991; Simon et al., 1992; Svartberg et al., 2003). The production of T is strongly influenced by numerous factors that can accelerate its age-related decline, such as concomitant disease states, comorbidities, drug therapies, and multiple environmental influences (Feldman et al., 2002). Relating to this, it has been suggested an age-independent time trend according to which males of the second half of the 1900s had higher T-levels than their contemporaries. In 2007, Anderson et al. evaluated 5,350 male participants of four large population-based surveys in Denmark, in which serum levels of the total T, of sex hormone-binding globulin (SHBG) and of free T, estimated by the Vermeulen equation (Vermeulen et al., 1999), were quantified in a 20-year period between 1982 and 2001 (Andersson et al., 2007). Authors reported a significant age-independent decline in serum T-levels and a significant increase of SHBG levels, both concurring to a reduction of free T. Authors speculated assigning these trends to a concurrent increase in body mass index. In the same year, Travison et al. provided almost overlapping results from a re-analysis of data collected during the Massachusetts Male Aging Study (MMAS), a community-based random sample observational survey on 1,709 non-institutionalized 40–70-year-old men, conducted from 1987 to 1989 in the urban area around Boston, Massachusetts (Feldman et al., 1994; Travison et al., 2007). Authors described an estimated cross-sectional decline of serum T-levels by .4% per year, whose extent was barely modified by possible confounding factors such as weight gain, smoking habits, and concurrent therapies. Also, bioavailable T, obtained by the correction for SHBG levels the other confounding covariates, showed a significant decrease by 1.3% per year (Travison et al., 2007). Another study in 2013 was conducted by Perheentupa et al. in a Scandinavian cohort of 3,271 men in the age range 25–74 years, pertaining to three surveys on Finnish population of 1972, 1977, and 2002 (Perheentupa et al., 2013). Authors documented a consistent lowering of serum T-levels across decades in subjects of the same age, whose trend was not affected by correction for BMI. In parallel, Mazur et al., originally aiming to study the possible health consequences of dioxin exposure during the Vietnam War, evaluated 991 men from the Air Force Health Study (AFHS) (Mazur et al., 2013). Across the six cycles of evaluation, authors reported a decrease in mean serum T-levels from 638 ng/dL in 1982 to 431 ng/dL in 2002, nearly doubling the estimated reduction due to the aging effect, even recognizing a parallel trend toward weight gain as a possible confounding factor. More recently, Chodick et al. analyzed serum T-levels in 102,334 male subjects of Middle Eastern origin, aged 13–80 years old, in the period 2006–2019 (Chodick et al., 2020). In spite of a non-significant difference in terms of BMI and participant’s age across the different periods in the study duration, a highly significant age-independent decline in serum T-levels was reported. Finally in 2021, Lokeshwar et al. evaluated 4,045 men from the National Health and Nutrition Examination Surveys, investigating serum T-levels during the period 1999 to 2016 (Lokeshwar et al., 2021). Even after normalization for BMI, authors reported a significant reduction in mean T-levels in the evaluations within the period 2011–2016 compared to period 1999–2000. Whilst acknowledging some possible environmental or life-style influence on secular trend toward T-level reduction, none of the aforementioned studies considered any possible role of environmental temperature, substantially leaving a great interpretative void to this evidence.

#### Effect of environmental temperature on birth rate

There are a few available studies which address the possible link between the progressive reduction in birth rate and global warming.

In 2018, Barreca et al. conducted a study aimed to estimate the effects of heat waves on birth rates (Barreca et al., 2018). The authors focused on the state birth registry of the United States from 1931 to 2010, correlating the state-by-month birth count with weather data from the National Climatic Data Center’s Global Historical Climatology Network, in order to address the possible effect of the daily ambient temperature on the birth rate in the following months. By using a previously validated regression model (Barreca, 2012), the authors showed that every additional day with an average ambient temperature above 26.7°C (80°F) associates with a subsequent decrease in the birth rate after 8, 9, and 10 months, by approximately 06%, 40%, and 21%, respectively, suggesting that the critical period for heat exposure is right before conception. Interestingly, a rebound increase in birth rates was observed 11, 12, and 13 months after the heat wave (Barreca et al., 2018). This evidence, joined with the lack of any significant influence of temperature exposure at months 1, 2, or 3, is not in agreement with the aforementioned data according to which sperm count decline occurs 2–3 months after the heat wave (Santi et al., 2018; Zhou et al., 2020; Xiao L. et al., 2022). Acknowledging seasonality in births, with an acme in August and a nadir in April, the authors account for the possible role of December holidays in the displacement in conceptions and subsequent deliveries in September and October, without addressing the involvement of any male factor. Interestingly, evaluating the effects of each additional day with >26.7°C on the birth rate at 9 months by decade, 1940s, 1950s, and 1960s’ data were more pronounced than to the 2000s (featured by a reduction of, respectively, 0.6% compared to 0.2%). In order to explain this trend, the authors accounted for possible errors in temperature assignment in early years or, more likely, to the diffusion of air conditioners from the 1950s onward (Barreca et al., 2018).

More recently, Jensen et al. addressed the possible influence of ambient temperatures on total fertility rates, in countries across the world, in a wide period between 1860 and 1980 (Jensen et al., 2021). The authors included in the analysis 65 nations, large enough represent the world population and, at the same time, relatively small in order to avoid major size-related bias in temperature assignment. For the analysis, the authors considered the respective annual total fertility rate related with the corresponding annual maximum temperature and temperature amplitude, namely, the difference between maximum and minimum temperatures of the year. Overall, maximum temperatures negatively affected the total fertility rate in both the current and preceding generation since, at each 1°C of increase, a 3.6 and 3.9 reduction of children per mother was observed. Differently, temperature amplitudes appeared to positively affect the total fertility rate by 1.5 and 1.4 children per mother. This evidence was even more emphasized in those countries less affected by fertility interventions policies and where a positive correlation between maximum temperatures and temperature amplitudes was observed, showing a reduction in the total fertility rate of 5.1 children per mother. The authors concluded by supporting the existence of a seasonal-based variation of fertility, particularly in populations which featured monthly maximum temperatures greater than 15°C–20°C. Differently, a positive or no effect was found for people living in colder climates experiencing elevated season temperatures (Jensen et al., 2021).

## Conclusion

**FIGURE 3:**
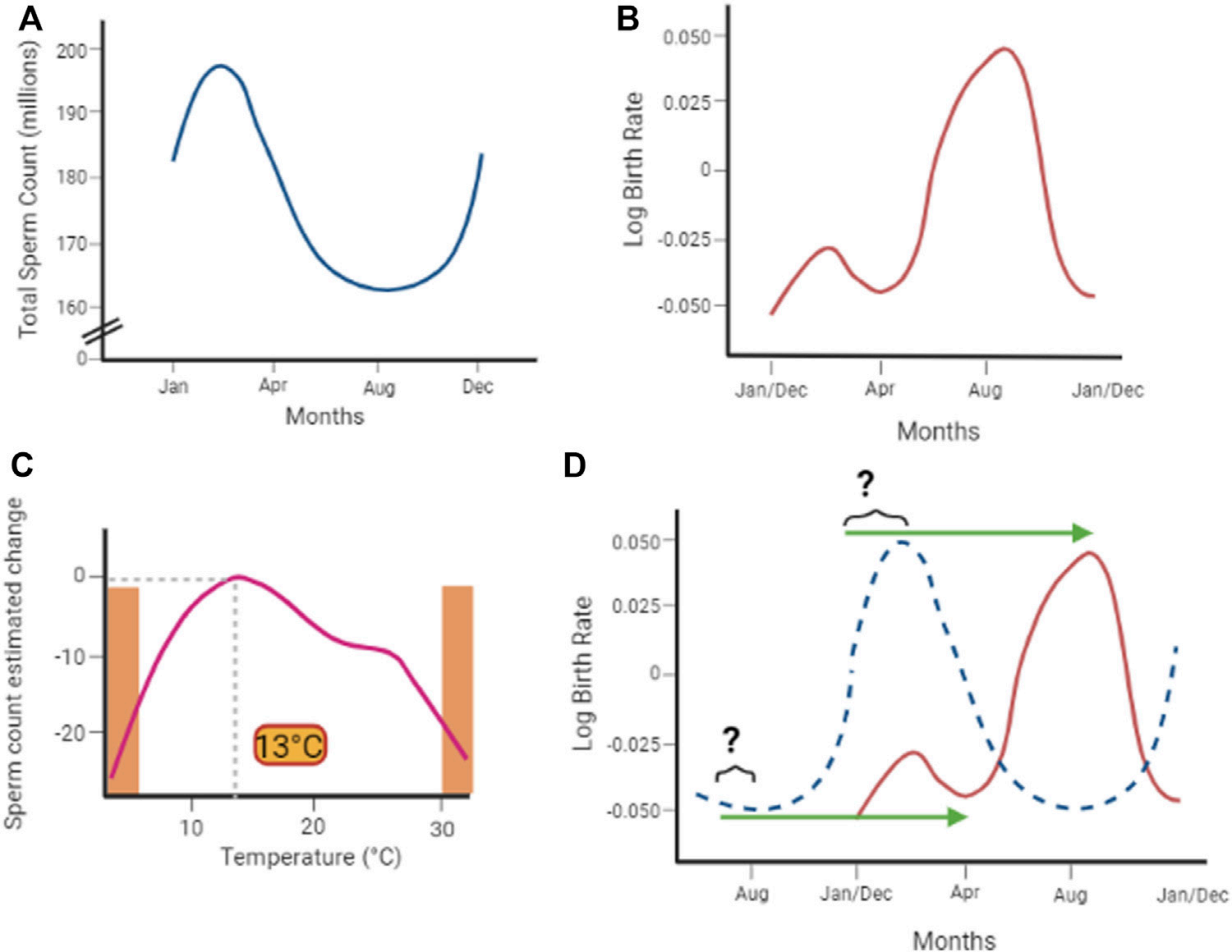
**(A)** Available epidemiological studies describe a seasonal trend of semen sperm count and sperm parameters, highly correlated with the environmental temperature. **(B)** Ambient temperature of 13°C associates with the best spermatogenetic performance, which linearly decreases moving away from this optimum and sharply drops at extreme temperatures (orange areas in the plot. Adapted from Zhou et al., 2020). **(C)** Also, the birth rate shows a seasonal trend (adapted from Barreca et al., 2018). However **(D)**, even considering a 9-month-delay effect associated with gestation (green arrows), there is poor consistency with seasonal variation sperm count (dotted blue curve).

The increase of environmental temperatures associated with greenhouse gas accumulation is a recognized phenomenon having a major impact on the health of both animals and humans, in current and future generations. In this regard, because of its anatomical location and tissue architecture optimized to warrant an overall scrotal temperature of 2°C–4°C lower than the core body temperature, the function of the testis is thoroughly influenced by environmental temperature. A large amount of evidence, summarized in Table 1, reports the detrimental effects of acute heat stress on testis function, particularly on the spermatogenetic and steroidogenetic process, in both animal and human models. Most of these effects are reversible over time and the proposed molecular mechanism generally relies on the temperature-dependent impairment of cells redox balance, followed by disruption of the cell cycle and germ cell apoptosis. Available epidemiological studies generally agree with a seasonal trend of human sperm count and sperm parameters (Figure 3A). These oscillations are highly correlated with the environmental temperature, and an ambient temperature of 13°C associates with the best spermatogenetic performance (Figure 3B). Available studies also show that the birth rate has a seasonal trend and is significantly affected by extreme temperatures (Figure 3C). However, there is a substantial lack of data that can support a causal link between the progressive reduction of the global birth rate with the worsening of seminal parameters and the increase of environmental temperature.

**TABLE 1:** Description of the studies evaluating the effect of heat stress challenge on spermatogenesis and on steroidogenesis in animal and human models.

Effect of the heat-stress challenge on spermatogenesis in animal models			
Reference	Model	Heat Stress	Results
Rockett et al. (2001)	Male C57BL/6 mice	Scrotal heat exposure to 43 °C for 20 min (lower half of the torso was submerged in a water-bath)	- Diffused vascular degeneration in seminiferous tubule cell populations after 8 h of exposure to 43 °C
			- Germ cell nuclei had highly condensed chromatin/apoptotic bodies
		Controls: water bath at 33 °C for 20 min	- Germ cell degeneration after 16 h from thermal stress
			- Apoptosis temporally correlated with the expression of stress-inducible Hsp70-1 and Hsp70-3 proteins in spermatocytes
Cai et al. (2011)	Male ICR mice	Scrotal heat exposure to 43 °C for 30 min (lower half of the body was submerged in a water-bath)	- ↓ expression of the tight-junction components occludin, claudin-3, and ZO-1 for 24–48 h after heat shock
		Controls: room temperature	- ↑ claudin-11 during the 3 days following thermal stress
Alves et al. (2016)	Healthy white Dorper rams	Scrotal insulation for 72 h through the use of heating bags	- ↓sperm motility and morphology after 21 days from heat stress
		Controls: not submitted to scrotal insulation	
Shadmehr et al. (2018)	Adult NMRI male mice	Lower half of the body was submerged in a water-bath at 42 °C for 30 min	- Impaired maturation of the germ cell population
			- ↓ testicular weight and the quality/quantity of epididymal spermatozoa
		Controls: water-bath at 33 °C for 30 min	- ↑levels of anti-oxidative enzymes
			- ↑ PCNA and Cyclin D3
Garcia-Oliveros et al. (2020)	Healthy *Nellore* breed bulls	Thermostated scrotal bag maintained at ∼32 °C for 96 h	After 14 days
		Controls: ∼30 °C for 96 h	- ↑ lipid membrane peroxidation levels of spermatozoa
			- ↓ mitochondrial membrane potential, motility parameters, and the plasma membrane integrity of the sperm
			After 28 days
			- ↑ sperm DNA fragmentation index
Wu et al. (2020)	Adult male ICR mice	Immersed in a water-bath at 43 °C for 30 min	- ↓ AKAP4, ODF1, GAPDHS, SPESP1, and ACTRT2
		controls:33 °C for 30 min	
Rizzoto et al. (2020)	C57BCL/6 elite male mice	Distal third of the body (including the scrotum) immersed at 40 °C for 20 min in water	- ↑ expression of the StAR gene 14 days after heat stress
		Controls: 30 °C for 20 min	
Hirano et al. (2022)	Male C57BL/6 J mice were subjected to operations of artificial cryptorchidism	Scrotal temperature measured with a transponder in the scrotal sac/peritoneal cavity	- Arrest of spermatogenesis at the primary spermatocyte, late spermatocyte, or round spermatid stage at 38 °C, 37 °C, and 36 °C, respectively
Jeremy et al. (2022)	Healthy adult Swiss albino male mice	Single heat stress treatment at 43 °C for 15 min (the lower parts of the body were submerged in a thermostatic water bath)	- ↓ germ cell proliferation
		Controls: immersed at 25 °C for 15 min	
Effect of the heat-stress challenge on spermatogenesis in human models			
Figà-Talamanca et al. (1996)	Taxi drivers in Rome	Reduced heat dissipation	- Testosterone levels do not vary with years in the profession
	Controls: healthy subjects		
Saikhun et al. (1998)	Healthy men	Sauna at a temperature of 80°C–90 °C for 30 min/day for 2 weeks	- ↑ scrotal temperature during and after sauna exposure
			- ↓ semen volume, sperm concentration, and total sperm count in the first week of exposure
			- ↓ VAP, VCL, and ALH (sperm movement parameters) after 2 weeks of sauna
Bujan et al. (2000)	Healthy men	Cutaneous thermocouples attached to the skin on the anterior face of the scrotum monitoring by walking outside for 40 min and driving a car for 160 min	- ↑ scrotal temperature in driving posture after 2 h of driving
Shefi et al. (2007)	Infertile men	wet heat exposure (hot tubs, hot baths or whirlpool baths) above 37 °C for ≥30 min/week during ≥3 months prior to presentation	- Wet testis hyperthermia has a reversible negative effect on sperm motility
Garolla et al. (2013)	Normo-zoospermic volunteers	Two sauna sessions/week for 3 months, at 80°C–90 °C, each lasting 15 min	- Sauna exposure induces significant but reversible impairment of spermatogenesis, and alteration of sperm parameters, mitochondrial function, and sperm DNA packaging
Wu et al. (2020)	Men who had at least one child (aged 22–50 years)	Testicular warming in a 43 °C water bath for 30 min a day for 10 consecutive days	- ↓ 60 proteins evaluated 6 weeks after heat stress, involved in spermatogenesis, fertilization, or other aspects of reproduction
Fraczek et al. (2022)	Fertile (fathered at least one child) and infertile (failed natural conception after 12 months) men	- A group of professional drivers	- ↑ sperm with DNA fragmentation in all the studied subgroups vs*.* fertile men
		- A group of infertile men with varicocele	
		- A group of infertile men not exposed to prolonged genital heat stress	- ↑ oxidative stress scavenging in professional drivers/infertile men with varicocele
		- A group of healthy fertile men not exposed to prolonged genital heat stress as controls	
Effect of the heat-stress challenge on steroidogenesis in animal models			
Alves et al. (2016)	Healthy white Dorper rams	Scrotal insulation for 72 h using heating bags	- ↓serum T in rams undergone scrotal insulation vs*.* controls
		Controls: not submitted to scrotal insulation	
Li et al. (2016)	Adult male Sprague–Dawley rats	Lower one-third of the body was sub merged once in a 43 °C water-bath for 30 min	- ↑ expression of cell cycle proteins in proliferating Leydig cells
		Controls: room temperature for 30 min	- ↓ serum and testicular testosterone concentrations
			- ↓testosterone biosynthesis by the suppressed expression of steroidogenic enzymes (CYP17 and StAR)
Shadmehr et al. (2018)	Adult NMRI male mice	Lower half of the body was submerged in a water-bath at 42 °C for 30 min	- Inadequate synthesis of testosterone
		Controls: water bath at 33 °C for 30 min	
Rizzoto et al. (2020)	C57BCL/6 elite male mice	Distal third of the body (including the scrotum) immersed at 40 °C for 20 min in water	- ↑ testosterone support for sustaining germ cell renewal
		Controls: 30 °C for 20 min	
Jeremy et al. (2022)	Healthy adult Swiss albino male mice	Single heat stress treatment at 43 °C for 15 min (the lower parts of the body were submerged in a thermostatic water bath)	- ↓circulating levels of testosterone
		controls: immersed at 25 °C for 15 min	
Effect of the heat-stress challenge on steroidogenesis in human models			
Figà-Talamanca et al. (1996)	Taxi drivers in Rome		- Testosterone levels do not vary with years in the profession
	Controls: healthy subjects		
Garolla et al. (2013)	Normo-zoospermic volunteers	Two sauna sessions/week for 3 months, at 80°C–90 °C, each lasting 15 min	- No significant change in sex hormones

The opportunity to conduct accurate association studies on this topic is hindered by numerous factors. First of all, the accessibility of precise retrospective data in the baseline period 1850–1900s. In addition, the progressive variation of environmental temperature associates with parallel variations of lifestyle and nutrition patterns through the modification of local ecosystems, indirectly modifying the health state of the population under examination. Furthermore, the understanding of the effective individual thermoregulation capacity would benefit from the precise measurement of scrotal temperature. However, this evidence is essentially lacking or pertains to studies performed in a poorly representative cohort. Finally, from a mechanistic point of view, there is a substantial lack of data able to link the production of ROS, which is a relatively non-specific tissue response to heat stress, to specific processes in defined cell populations, such as the germ line cell apoptosis. The widening of all these elements represents the current challenge of estimating the impact of environmental temperature on health, which is one of the major scientific commitments for the next future.
